# Interventions to Support Integrated Psychological Care and Holistic Health Outcomes in Paediatrics

**DOI:** 10.3390/healthcare5030044

**Published:** 2017-08-16

**Authors:** Roz Shafran, Sophie D. Bennett, Mhairi McKenzie Smith

**Affiliations:** UCL Great Ormond Street Institute of Child Health, 30 Guilford St., London WC1N 1EH, UK; Sophie.bennett.10@ucl.ac.uk (S.D.B.); mhairi.mckenzie.16@ucl.ac.uk (M.M.S.)

**Keywords:** psychological intervention, goal based outcomes, paediatrics

## Abstract

There are strong calls from many national and international bodies for there to be a ‘holistic’ and integrated approach to the understanding and management of psychological and physical health needs. Such holistic approaches are characterized by the treatment of the whole person, taking into account mental and social factors, rather than just the symptoms of a disease. Holistic approaches can impact on mental and physical health and are cost-effective. Several psychological interventions have demonstrated efficacy in improving holistic health outcomes, for example Cognitive Behaviour Therapy, Behavioural Therapies and Problem Solving Therapies. They have shown to impact upon a wide range of outcomes, including psychological distress, pain, physical health, medication adherence, and family outcomes. There is increasing recognition that the holistic goals of the child and family should be prioritised, and that interventions and outcomes should reflect these goals. A focus on holistic goals in therapy can be achieved through a combination of personalised goal-based outcomes in addition to symptom-based measures.

## 1. The Benefits of Integrated Psychological Care

There is an unfortunate and longstanding tradition of considering the mind and body as separate and distinct entities. Such separation or ‘dualism’ is typically attributed to the philosopher René Descartes (1596–1650), although there is debate as to his exact position regarding the implications of this for health [1]. It has taken until the late 20th and early 21st Centuries for there to be strong calls for there to be a ‘holistic’ and integrated approach to the understanding and management of psychological and physical health needs. Such holistic approaches are characterized by the treatment of the whole person, taking into account mental and social factors, rather than just the symptoms of a disease.

There are several reasons for the movement towards an integrated approach to psychological care and physical health. First and foremost, having a long term physical condition greatly increases the risk of experiencing psychological distress [2,3,4]. Two of the most common and typical childhood chronic illnesses associated with elevated psychological disorders are epilepsy and Type 1 diabetes. In the classic community Isle of Wight study, 29% of children with epilepsy had at least one psychological disorder, over four times the rate in the general population [5]. In diabetes clinics, 47% of children were found to develop significant psychological needs at 10-year follow-up [6].

The mechanisms of this relationship are not fully understood; physical and psychological conditions may share the same pathology [7], or the stress associated with having a long-term condition and its treatment may affect psychological functioning [8]. In children, a long-term illness may impact on all domains of development and functioning across the age range including social development, separation from the family of origin, individuation, developmental tasks, resilience and having a sense of identity away from the illness [9,10,11,12].

In addition to the impact of a chronic illness on psychological functioning, psychological distress may impact upon the chronic illness. For instance, psychological distress may lead to poor treatment compliance, which exacerbates the physical condition, and impairs self-management and worsens long-term outcome [13]; depression in children with diabetes is associated with poorer blood sugar control [14] and in turn with later retinal damage [15]. Psychological disorders have been associated with increased seizure frequency in juvenile myoclonic epilepsy [16]. Overall, serious psychological disturbance has the same effect on life-expectancy as smoking, and more than obesity [17]. This has been partly attributed to psychological distress intensifying the effects of physical illness through physiological responses. For example, psychological distress may increase the production of stress hormones such as cortisol, which may undermine the immune system [17].

A second, less scientific but highly pragmatic reason for the move towards integrated healthcare is the recognition of the financial cost of having both a physical and psychological health need. In adults, Co-morbid mental health disorders increase costs for long term conditions (LTCs) by 45% per person [18]. This means that roughly £1 in every £8 spent on LTCs is connected to poor mental health, equating to £8–13 billion of NHS spending in England each year [18]. Psychological interventions have significant clinical and cost benefits. Meta-analyses indicate that psychological interventions reduce care costs by 20% [19], equating to £1235 per case, and that the cost of psychological treatment is more than offset by the resulting savings in physical healthcare costs [18]. Considering psychological distress in the context of chronic illness, cognitive behaviour therapy (CBT)-based interventions can improve treatment adherence, psychosocial adjustment, coping skills and quality of life for people with co-morbid LTCs, as well as reducing use of health care services [20,21]; including a psychological component in a breathlessness clinic for Chronic Obstructive Pulmonary Disease (COPD) in adults led to 1.17 fewer A&E presentations and 1.93 fewer hospital bed days per person in the six months after the intervention, which translated into savings of £837 per person [22].

Not only are there cost-savings in relation to having both a physical and mental health condition, but in addition, for children and young people, there are savings related to early intervention and reducing costs across the lifespan. For example, a child with conduct disorder at 10 subsequently costs the government roughly £100,000 more than other children throughout his/her lifespan [23]. Having a mental health disorder by the age of 16 is associated with 19% lower family income at age 23, equating to an estimated £388,000 across the lifespan [24]. Overall, there are high potential cost savings for treating young people with mental health disorders and LTCs since the strategies learnt in youth for reducing mental health difficulties can be applied throughout the lifespan with widespread clinical, social and occupational benefits [25].

## 2. Evidence for Interventions to Support Integrated Psychological Care

Whilst there has been extensive research on the development and evaluation of psychological interventions aimed specifically to treat emotional distress in young people [26], very few studies have examined their use in populations with a chronic illness [27]. There is ongoing research as to the nature of the distress that arises in relation to having a long-term condition; specifically, (a) whether the distress is qualitatively different from that seen in other contexts (i.e., anxiety or low mood in children who do not have a chronic illness); and (b) whether any differences are phenomenological in nature or whether they are fundamental, such that standard psychological treatments shown to work for young people with psychological needs in the absence of long term illness require significant adaptation—i.e., modification throughout the intervention to account for the presence of a chronic illness [28,29,30].

The debate about the degree of ‘adaptation’ cuts across the range of different psychological treatment approaches that are used to help people with emotional distress. This is because although there are important differences in psychological treatment approaches, they also have much in common. Psychological treatment approaches all aim to help the young person and/or their families better understand and manage their distress (or behaviour), participate in developmentally appropriate activities that they could not previously engage in and ultimately help the young person to function better in the world around them. Within the range of psychological treatments, some have more scientific support than others and hence are recommended by bodies such as NICE.

However, it is important to bear in mind the distinction between ‘evidence of absence’ in which there is evidence the psychological treatment is not helpful (or is harmful) and ‘absence of evidence’ in which there is insufficient research to be able to establish the effectiveness of the intervention. Examples of areas in which there is research regarding the use of psychological interventions include interventions for mental health, chronic pain, procedural pain/anxiety and medication adherence. Each of these areas are expanded on in turn below.

### 2.1. Mental Health

Systematic reviews have demonstrated that there is a lack of evidence regarding interventions for significant emotional distress in children and young people with a chronic physical illness [27]. The evidence that is available suggests that these children may benefit from cognitive behavioural interventions for depression and anxiety. Such interventions may improve psychological distress, although there is little evidence that they improve physical health outcomes in children [27].

Cognitive behavioural interventions centre around changing unhelpful thoughts and behaviours; specifically, they involve identifying and challenging unhelpful thoughts/thinking patterns; cognitive restructuring to change behaviour in response to these thoughts and practice of desirable behaviours. Whilst CBT interventions may be manualised, this does not equate to a rigid, ‘one-size fits all’ approach. Rather such interventions consider ‘flexibility with fidelity’ [31], in other words the core components of the intervention are followed, but therapists must consider the goals and outcomes of importance to the client and be responsive to their needs. Such goals may focus on wider holistic needs, for example, being able to visit friends’ houses, in addition to purely symptom-based measures (such as depression or anxiety severity).

Considering the issue of adaptation, some CBT interventions have been modified to account for the presence of a chronic illness, with illness-specific content (e.g., CBT for anxiety in young people with Inflammatory Bowel Disease [32]); whereas others have not altered the content [33]. Regarding integration of physical and psychological healthcare, whilst many studies did not adapt or modify the content of interventions to account for the presence of chronic illness, several CBT studies allowed for more flexible delivery through offering telephone sessions, or sessions at home/the medical treatment location.

One reason for reviews demonstrating a lack of evidence in this area may be that mental health clinicians are concerned about the safety of these interventions in a population with chronic illness. For example, one of the key elements of CBT for anxiety is exposure to the feared stimulus. Clinicians may be concerned about causing undue distress to a child who is already physically unwell, and potentially causing a worsening of the physical health symptoms, for example triggering an attack in a child with asthma or seizure in a child with epilepsy. A key element of ensuring integrated, holistic care in these interventions is through close liaison between the psychology/mental health team and the physical healthcare team, for example through joint assessments and integrated treatment plans, consulting with the paediatrician where appropriate. In addition, lack of evidence may reflect the inclusion criteria of reviews undertaken to date, which have focused on interventions aimed at reducing psychological distress. In addition to the studies explored in these reviews, it may be that some psychological interventions designed primarily to treat an issue other than emotional distress, for example those focused on medication adherence, may also affect psychological outcomes; however, research studies of these interventions generally do not investigate cohorts of children with elevated levels of emotional distress.

### 2.2. Chronic Pain

The close integration between physical and psychological healthcare is exemplified by the research on the management of the pain that often accompanies the physical health condition. Core Standards for Pain Management Services in the UK [34] stipulate that children with chronic pain should be offered psychological intervention in addition to pharmacological and physical treatments, owing to the well-established biopsychosocial models of chronic pain [35] and the large number of randomised controlled trials that convincingly demonstrate the positive impact of psychological intervention on pain and broader functioning [36,37]. Interventions for pain include CBT, relaxation training/biofeedback, and Exposure and Acceptance based therapies, such as Acceptance and Commitment Therapy (ACT [38]). Relaxation training involves teaching relaxation strategies/techniques such as progressive muscle relaxation, breathing techniques and/or imagery. In addition, biofeedback may be used, in which monitors are placed to record biological indicators of stress/anxiety, such as muscle activity, heart rate, or breathing rate. This is used to increase awareness of the stress response, and the effect of relaxation exercises on this response. ACT interventions promote the acceptance of negative thoughts or feelings (e.g., pain, negative emotions) and engagement in activities that are meaningful to the person, even when this may trigger negative thoughts/feelings. For example, in pain interventions, a child may be encouraged to partake in activities that are meaningful to them, even if they cause pain. The child then gradually distances themselves from pain and pain has a lesser impact on their behaviours and therefore life [39]. Psychological interventions for pain have been shown to have a positive effect on both pain and disability, but outcomes on psychological distress are less clear [37]. A review of psychological interventions for *parents* of children and young people with a chronic physical illness found good evidence for the effectiveness of interventions that included parents for reducing pain, but not other outcomes such as psychological distress, in children with chronic pain [40].

### 2.3. Procedural Pain, Distress and Anxiety

Distraction (for example through toys or games) and hypnosis have been demonstrated to reduce needle-pain and distress [41]. A recent review found no evidence to support the efficacy of educational interventions (preparation and information), CBT, parent coaching or virtual reality on needle related pain and distress [42]. However, this may be a separate issue from a needle phobia, in which a child or young person may worry and/or have significant fear in advance of a procedure. Phobias of procedures, including venepuncture, may be particularly impairing for those with chronic illness, and may affect physical health, through cancellations of operations, or poorer adherence to medical regimes. For example, in children with diabetes, a greater fear of needles has been associated with higher HbA1c levels and less frequent blood sugar monitoring [43]. One-session exposure interventions have demonstrated efficacy in treating phobias in adults, and their effectiveness has recently been demonstrated in a trial for blood-injection-injury phobia in children and young people [44].

### 2.4. Adherence

Several reviews have demonstrated the efficacy of psychological interventions for improving adherence to medications/compliance with a medical regime [45,46], which has implications for physical wellbeing. Interventions commonly include behavioural (interventions with a focus on behaviour change) or cognitive-behavioural, and may include family elements. Motivational interviewing, which uses conversation to help a person consider their ambivalence towards a particular behaviour (e.g., adhering to medication regimes), and ways in which that behaviour is in line with a person’s current values and future goals (e.g., being able to participate in sports) may be used with adolescents [47]. A meta-analysis [48] demonstrated medium effect sizes for behavioural and multi-component interventions, which involved a combination of one or more of social-support, social skills training, family therapy and a behavioural or educational component. Conversely, educational interventions alone and broader psychosocial interventions alone, which were focused around family functioning or crisis intervention but not on the adherence behaviour per se, only demonstrated small effect sizes. Technology-based interventions, such as games or glucose meters, did not demonstrate any significant effect overall; these were delivered without ongoing guidance or clinical support. As treatment adherence reduces from childhood to adolescence [49] and young people have stated a preference for technology based interventions [50], future research may benefit from enhancing efficacy of such interventions, perhaps through combining them with clinician support.

### 2.5. Wider Family Outcomes

Family based interventions have demonstrated efficacy across a range of childhood chronic illnesses. In addition to positive effects on child outcomes, Problem Solving Therapy which includes parents has been demonstrated to improve parental mental health [40]. Problem Solving Therapy [51] involves teaching a person a structured way to identify specific problems causing them distress, identify potential solutions, and evaluate the outcome of the chosen solution, thereby promoting self-efficacy, although interventions targeted directly at parental mental health have had inconclusive findings [40]. Other family based interventions include family therapy (FT) and Multisystemic Therapy (MST). Such interventions consider that social relationships between people and contexts cause and maintain problematic behaviour, and the focus of the intervention is therefore on these relationships; MST is an intensive home-based therapy considering the family and relationships with wider systems (such as school). The overall efficacy of family based therapies on parent and child outcomes is unclear [40].

## 3. Holistic Health Care Outcomes and what Matters to Patients

The above review of the literature makes it clear that there are a wide range of health outcomes that are amenable to psychological intervention, ranging from psychological distress to physical health outcomes, or adherence to a medical regime. However specific studies/interventions often focus on one primary outcome and this may not be reflective of the outcomes of primary importance to patients or families. For example, the little research available on the treatment of psychological distress in the context of a long-term condition has typically used a specific, symptom-based measure to assess the impact of the intervention. Such measures often do not capture the broader holistic goals of therapy in terms of helping the young person function better in the world around them and continue along the developmental trajectory; it may be more meaningful for a young person to be able to attend after-school activities, than reduce their symptom-count on a depression severity measure, for example. With the advent of targets to demonstrate the impact of our interventions on patients, the challenge is how to choose the correct metric to demonstrate the benefit to patients and their families. Symptom-based measures are clearly necessary but they are also likely to be insufficient [52] which is why psychological services also use goal-based measures to allow for personalisation of treatment outcomes [53]. In addition, the specific intervention chosen should reflect the evidence-base for the goals of the child and family, considering the outcomes that are most important to them. For example, if the primary goal is pain reduction, then an ACT-based intervention may be appropriate, but if it is to reduce anxiety, then a CBT intervention (as recommended by NICE [54]) may be more effective.

## 4. The Way Forward

Overall, this review highlights several psychological interventions for children and young people who have a chronic illness that have demonstrated efficacy across a range of areas, including pain, mental health, procedural anxiety, adherence to a medical regime and wider family outcomes. Integrating psychological care within the paediatric setting is therefore essential if we are to improve holistic patient outcomes. Adult studies also suggest that such an approach is cost effective. Young people and their families want this type of joined-up approach to their healthcare. At its very simplest, this may mean coordinating appointments with separate psychology and paediatric services or offering co-located services, to minimise travel and time burden. At its most sophisticated, it would mean having systems to identify psychological needs early in the process and to personalise the psychological intervention to both the individual and family characteristics, as well as the characteristics of the long-term condition [55]. Such personalisation would, by necessity, capture the outcomes that were most meaningful and important to the young person and the family as well as standardised outcomes that allow clinicians and researchers to benchmark progress and ensure minimisation of harm. We are living in exciting and changing times where, finally, the voice of the young person and the family is being prioritised Such personalisation through listening to the goals and aims of the family is the key to holistic health outcomes that reflect the full potency and benefit of an integrated healthcare approach.
